# Evolutionary and Expression Analysis of miR-#-5p and miR-#-3p at the miRNAs/isomiRs Levels

**DOI:** 10.1155/2015/168358

**Published:** 2015-05-05

**Authors:** Li Guo, Jiafeng Yu, Hao Yu, Yang Zhao, Shujie Chen, Changqing Xu, Feng Chen

**Affiliations:** ^1^Department of Epidemiology and Biostatistics, School of Public Health, Nanjing Medical University, Nanjing 211166, China; ^2^Shandong Provincial Key Laboratory of Functional Macromolecular Biophysics, Institute of Biophysics, Dezhou University, Dezhou 253023, China

## Abstract

We mainly discussed miR-#-5p and miR-#-3p under three aspects: (1) primary evolutionary analysis of human miRNAs; (2) evolutionary analysis of miRNAs from different arms across the typical 10 vertebrates; (3) expression pattern analysis of miRNAs at the miRNA/isomiR levels using public small RNA sequencing datasets. We found that no bias can be detected between the numbers of 5p-miRNA and 3p-miRNA, while miRNAs from miR-#-5p and miR-#-3p show variable nucleotide compositions. IsomiR expression profiles from the two arms are always stable, but isomiR expressions in diseased samples are prone to show larger degree of dispersion. miR-#-5p and miR-#-3p have relative independent evolution/expression patterns and datasets of target mRNAs, which might also contribute to the phenomena of arm selection and/or arm switching. Simultaneously, miRNA/isomiR expression profiles may be regulated via arm selection and/or arm switching, and the dynamic miRNAome and isomiRome will adapt to functional and/or evolutionary pressures. A comprehensive analysis and further experimental study at the miRNA/isomiR levels are quite necessary for miRNA study.

## 1. Introduction

MicroRNAs (miRNAs) have been widely studied as a class of well-conserved negative regulatory molecules. They play an important role in biological processes by regulating gene expression at the posttranscriptional level [1, 2]. As endogenous small noncoding RNAs (ncRNAs) (~22 nt), miRNAs are generated from the cleavage of primary miRNAs (pri-miRNAs) and precursor miRNAs (pre-miRNAs) by Drosha and Dicer cleavage [3–5]. miRNA may be generated from 5p or 3p arm of pre-miRNA, and the selection is believed to be influenced by hydrogen-bonding selection [6]. Based on the typical miRNA genesis, one arm can produce abundant active mature miRNAs, while another arm can produce rare and inactive miRNAs^∗^ (miRNA star, also ever named passenger strand). However, increasing evidence indicates that both arms can generate mature miRNAs under specific developmental stages or species [7–13]. Indeed, many pre-miRNAs have been reported to yield two kinds of mature miRNAs, although the two products, miR-#-5p and miR-#-3p, may vary in expression levels. The term given to this dynamic selection and expression is “arm switching” [8, 14]. Evolutionary analysis demonstrates that both miR-#-5p and miR-#-3p are conserved, although the nondominant miRNA sequences are not well-conserved with dominant miRNA sequences [15]. Increasing reports indicate that the nondominantly expressed miRNA sequences may act as potential regulatory molecules with unexpectedly abundant expression levels [16–18].

Although the typical miRNA is annotated and studied as a single sequence, accumulating evidence suggests that multiple sequences with varied 5′ and/or 3′ ends or varied lengths have been detected from the miRNA locus. The annotated or canonical miRNA is only one specific member of the multiple sequences. These multiple sequences are termed miRNA variants, also named isomiRs [19–23]. The miRNA isoforms are mainly derived from imprecise cleavage by Drosha/Dicer and 3′ addition events through miRNA processing and maturation processes. RNA editing and single nucleotide polymorphisms (SNPs) also contribute to the generation of these multiple isomiRs [22]. The occurrence of multiple isomiRs is quite common, and each miRNA locus can be associated with these various miRNA isoforms [9, 19, 21, 23–30]. Despite the fact that both miR-#-5p and miR-#-3p are generated from the pre-miRNA and can form miRNA:miRNA duplex through nucleotide complementary base pairing, the two miRNA loci may yield various isomiR expression profiles and patterns [31].

This study aimed to explore the potential evolutionary and expression divergences and relationships between miRNAs from different arms of different/same pre-miRNAs. First, we characterized the origins and nucleotide compositions of all the annotated human miRNAs. Second, we performed evolutionary analysis on the common miRNAs among 10 typical vertebrates and then analyzed the nondominant miRNAs based on the pre-miRNAs. Finally, the expression analysis was performed in samples from female patients using published small RNA sequencing datasets. Because gender difference can affect isomiR expression profiles [32], and common variation affects various diseases and medically relevant characteristics in a sex-dependent manner [33], we selected female patients to analyze miRNA/isomiR expression profiles to avoid potential effects from gender difference. miRNA expression patterns were mainly estimated at the miRNA/isomiR levels, especially between homologous miRNAs and between miR-#-5p and miR-#-3p. This study provides insights on the arm selection and/or arm switching in miRNAs from the evolutionary and expression angles, which would partly be informative to understanding the dynamic miRNAome and isomiRome and to characterizing miRNA and isomiR expression profiles. Study from the isomiR level may be a necessary way to understand miRNA, especially for those isomiRs from ever termed passenger stand, which will contribute to further explore miRNA biogenesis and function.

## 2. Materials and Methods

### 2.1. Source Data and Primary Analysis

According to the evolutionary taxa and numbers of known miRNA genes, 10 vertebrate species were selected:* Petromyzon marinus* (pma, Agnathostomata),* Danio rerio* (dre, Pisces),* Xenopus tropicalis* (xtr, Amphibia),* Anolis carolinensis* (aca, Lepidosauria),* Gallus gallus* (gga, Aves),* Equus caballus* (eca, Mammalia, Laurasiatheria),* Bos taurus* (bta, Mammalia, Ruminantia),* Monodelphis domestica* (mdo, Mammalia, Metatheria),* Mus musculus* (mmu, Mammalia, Rodentia), and* Homo sapiens* (hsa, Mammalia, Primates, Hominidae). All the pre-miRNAs and miRNAs were retrieved from the miRBase database (Release 20.0, http://www.mirbase.org/) [34].

Location information of miRNA on pre-miRNAs was obtained according to the annotations in the miRBase database. Specifically, miRNA generated from 5p arm of pre-miRNA was named miR-#-5p (# indicated the detailed miRNA name, such as miR-100), and miRNA generated from 3p arm of pre-miRNA was named miR-#-3p. If there is no existing annotation, the detailed location distributions were determined using self-developed scripts. Many miRNAs may be generated from multicopy pre-miRNAs, and herein we only presented the detailed isomiR expression profiles based on location of the first pre-miRNA. In the study, miR-#-5p and miR-#-3p were defined as miRNA pairs generated from the 5p and 3p arm of pre-miRNA, respectively, and 5p-miRNA and 3p-miRNA were defined as the miRNAs generated from 5p or 3p arm of different pre-miRNAs.

### 2.2. Evolutionary Analysis of miRNAs in Ten Test Vertebrates

Known annotated miRNAs from ten vertebrates were comprehensively surveyed for common miRNA members using self-developed scripts. These miRNAs were further classified based on the unit of miRNA gene family because many miRNAs could belong to the same gene family based on homologous sequences with high sequence similarity. Those pre-miRNAs that were not comprehensively annotated (miR-#-5p or miR-#-3p was not simultaneously annotated based on limited studies), unannotated miRNA sequences, were predicted and obtained from consensus sequences using pre-miRNAs and known human miRNAs. The main reasons were as follows: (1) human miRNAs have been widely studied, and most miR-#-5p and miR-#-3p are reported and annotated; (2) most miRNAs are phylogenetically well-conserved across different animal species, and well-conserved consensus sequences are easily obtained using sequence alignment analysis; (3) although the miR-#-5p and miR-#-3p show different levels of evolutionary divergence, both of them are conserved; (4) according to the known miRNA sequences and pre-miRNAs, the detailed miR-#-5p and miR-#-3p sequences can be collected. The shared miRNAs were aligned using Clustal X 2.0 multiple sequence alignment [35]. Nucleotide divergence was analyzed using MEGA 5.10 software [36] and DnaSP 5.10.01 software [37]. Simultaneously, nucleotide diversity (*π*), haplotype diversity (Hd), and average number of nucleotide differences (*k*) for the miRNAs from different animal species were calculated using DnaSP software as special miRNA populations [38]. Evolutionary patterns were estimated based on nucleotide divergence across the ten animal species using percentage of nucleotide substitutions (transition and transversion) and insertions/deletions in each position. The reference nucleotide was denoted as human miRNA. Based on the potential length difference between miRNAs in different species, we only analyzed the core sequences and not the terminus nucleotides with deficiency (these nucleotides were mostly derived from length differences). Nucleotide divergence patterns were further estimated between 5p-miRNA and 3p-miRNA and between miR-#-5p and miR-#-3p.

In order to track the evolutionary history of pre-miRNAs and miRNAs from the different arms, especially between homologous miRNAs, phylogenetic trees of pre-miRNAs were reconstructed using the neighbor-net method [39] in SplitsTree 4.10 [40], and networks of miRNAs were defined based on Jukes-Cantor model and Network 4.6.1.1 (http://www.fluxus-engineering.com/) using the median-joining (MJ) method. Also, the free energies of some pre-miRNAs were estimated through the RNAfold WebServer (http://rna.tbi.univie.ac.at/cgi-bin/RNAfold.cgi) [41, 42].

### 2.3. Analysis of the miRNA/isomiR Expression Levels Using Public Sequencing Datasets

In order to understand the expression patterns of miR-#-5p and miR-#-3p pairs, we analyzed them at the miRNA/isomiR levels using small RNA sequencing datasets generated by The Cancer Genome Atlas (TCGA) pilot project established by the NCI and NHGRI. Information about TCGA and the investigators and institutions constituting the TCGA research network can be found at http://cancergenome.nih.gov/. Available small RNA sequencing datasets associated with the three kinds of women's diseases including breast cancer (BRCA), ovarian serous cystadenocarcinoma (OV), uterine corpus endometrial carcinoma (UCEC), and their respective control samples were selected to investigate miRNA expression patterns at the miRNA/isomiR levels (see Table S1 in Supplementary Material available online at http://dx.doi.org/10.1155/2015/168358). We also conducted expression analysis in the three kinds of women's diseases dataset of some miRNAs (especially homologous miRNAs) identified from our evolutionary analysis. All of these high-throughput sequencing datasets were generated on Illumina HiSeq sequencing platform.

Reads per million (RPM) were used to estimate the relative expression levels, and relative expression rate (percentage) in the miRNA locus was used to assess the isomiR expression patterns across different samples. In order to track relative expression levels of miRNA/isomiR and reduce potential sequencing errors/mapping procedures, only those abundant miRNAs/isomiRs were selected to perform the analysis using larger sample sizes. The abundant expression and larger sample sizes could reduce error. Further, functional analysis was performed between miR-#-5p and miR-#-3p and between canonical miRNA sequences and their 5′ isomiRs (with the novel 5′ ends and seed sequences). According to the seed sequences, target mRNAs were predicted and obtained from TargetScan program (http://www.targetscan.org/).

### 2.4. Statistical Analysis

Data were evaluated using paired *t*-test (length distributions between miR-#-5p and miR-#-3p), Student's *t*-test (length distributions between 5p-miRNA and 3p-miRNA), Chi-square test (nucleotide compositions between different miRNAs from 5p or 3p), Wilcoxon signed-rank test (nucleotide divergence pattern between miR-#-5p and miR-#-3p), and Spearman correlation test (nucleotide divergence between miR-#-5p and miR-#-3p and homologous miRNAs). Differences were considered statistically significant if the *P* value is less than 0.05. All tests were two-tailed and conducted using Stata software (Version 11.0).

## 3. Results 

### 3.1. Primary Analysis of Human miR-#-5p/miR-#-3p and 5p-miRNA/3p-miRNA

**Figure 1:**
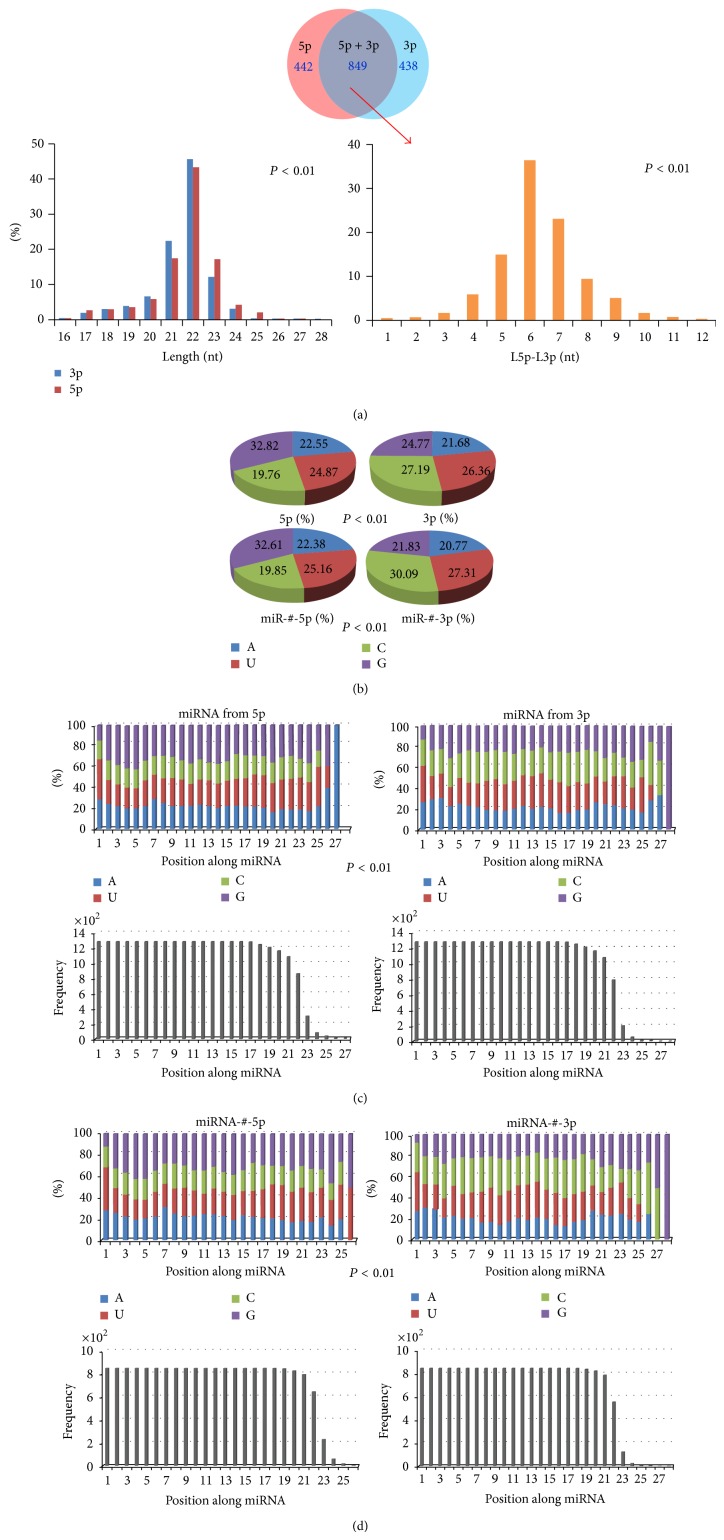
Primary analysis of human miRNAs according to their locations on pre-miRNAs. (a) Location and length distributions of human miRNAs. There are 849 pairs of miRNAs that are characterized as miR-#-5p and miR-#-3p from the same pre-miRNAs. Despite the fact that 22 nt is predominant length, the length distributions of 5p-miRNA and 3p-miRNA are highly variable (*t* = −2.68, *P* < 0.01). The frequency distribution of *D*-value (difference value) of miR-#-5p and miR-#-3p indicates that the two arms of pre-miRNA are likely to generate different miRNAs with different lengths (*t* = 6.01, *P* < 0.01). (b) Nucleotide compositions between 5p-miRNA and 3p-miRNA, miR-#-5p and miR-#-3p. Guanine (G) is the most predominant nucleotide in 5p-miRNA and miR-#-3p (more than 32%), while moderate distributions of the four nucleotides can be detected in 3p-miRNA and miR-#-3p. The two kinds of miRNAs are likely to have different nucleotide compositions (*χ* ^2^ = 400.02, *P* < 0.01). (c) Difference in nucleotide compositions based on the position along miRNA is detected between 5p-miRNA and 3p-miRNA (*χ* ^2^ = 656.70, *P* < 0.01). The frequency distributions of nucleotides in each position are also presented here. (d) The difference in nucleotide compositions based on position along miRNA is detected between miRNA-#-5p and miRNA-#-3p (*χ* ^2^ = 813.57, *P* < 0.01). The frequency distributions of nucleotides in each position are also presented here.

There were 2,578 annotated human mature miRNAs in the miRBase database (Release 20.0). A total of 1,291 miRNAs were characterized from the 5p arms of pre-miRNAs, while the others were characterized from the 3p arms. Of these, 849 pairs were identified as miR-#-5p and miR-#-3p from the same pre-miRNAs. Both 5p-miRNA and 3p-miRNA or miR-#-5p and miR-#-3p had different length distributions (5p-miRNA, 21.67 ± 0.04, 3p-miRNA, 21.51 ± 0.04, *t* = −2.68, *P* < 0.01; miR-#-5p, 22.08 ± 0.04, miR-#-3p, 21.72 ± 0.04, *t* = 6.01, *P* < 0.01, Figure 1(a)). 5p-miRNA and 3p-miRNA showed different nucleotide compositions (*χ* ^2^ = 400.02, *P* < 0.01, Figure 1(b) and Table 1). Guanine (G) was more predominant in 5p-miRNA (more than 32.82%) than in 3p-miRNA (24.77%). The predominant nucleotide in 3p-miRNA was cytosine (C) (27.19%), which was present at 19.76% in 5p-miRNA (Figure 1(b)). The presence of G, including double (GG), triple (GGG), and fourfold (GGGG) nucleotides, showed larger divergence between miRNAs from different arms (Figure 1(b) and Table 1). Similarly, the nucleotide composition was varied between miR-#-5p and miR-#-3p (Figure 1(b) and Table 1). Significant differences in the continuous nucleotide compositions could be detected between 5p-miR and 3p-miRNA and between miR-#-5p and miR-#-3p (Table 1). Compared to the total nucleotide compositions, nucleotides in each position along miRNA also showed significant difference between 5p-miR and 3p-miRNA and between miR-#-5p and miR-#-3p (*χ* ^2^ = 656.70, *P* < 0.01, Figure 1(c); *χ* ^2^ = 813.57, *P* < 0.01, Figure 1(d)), although the nucleotides 2–8, termed “seed sequences” of the miRNAs, did not display nucleotide bias.

### 3.2. Evolutionary Patterns of miR-#-5p/miR-#-3p and 5p-miRNA/3p-miRNA across Species

There were 31 miRNAs gene families (contain 43 miRNA members) shared by the 10 test animal species (Table S2). They may be composed of two or more members with high sequence similarity, but these members were not always shared by the 10 species. The common miRNA might have different number of pre-miRNAs (also termed multicopy pre-miRNAs) in different species and even have different number of homologous miRNAs (Figure S1).

**Figure 2:**
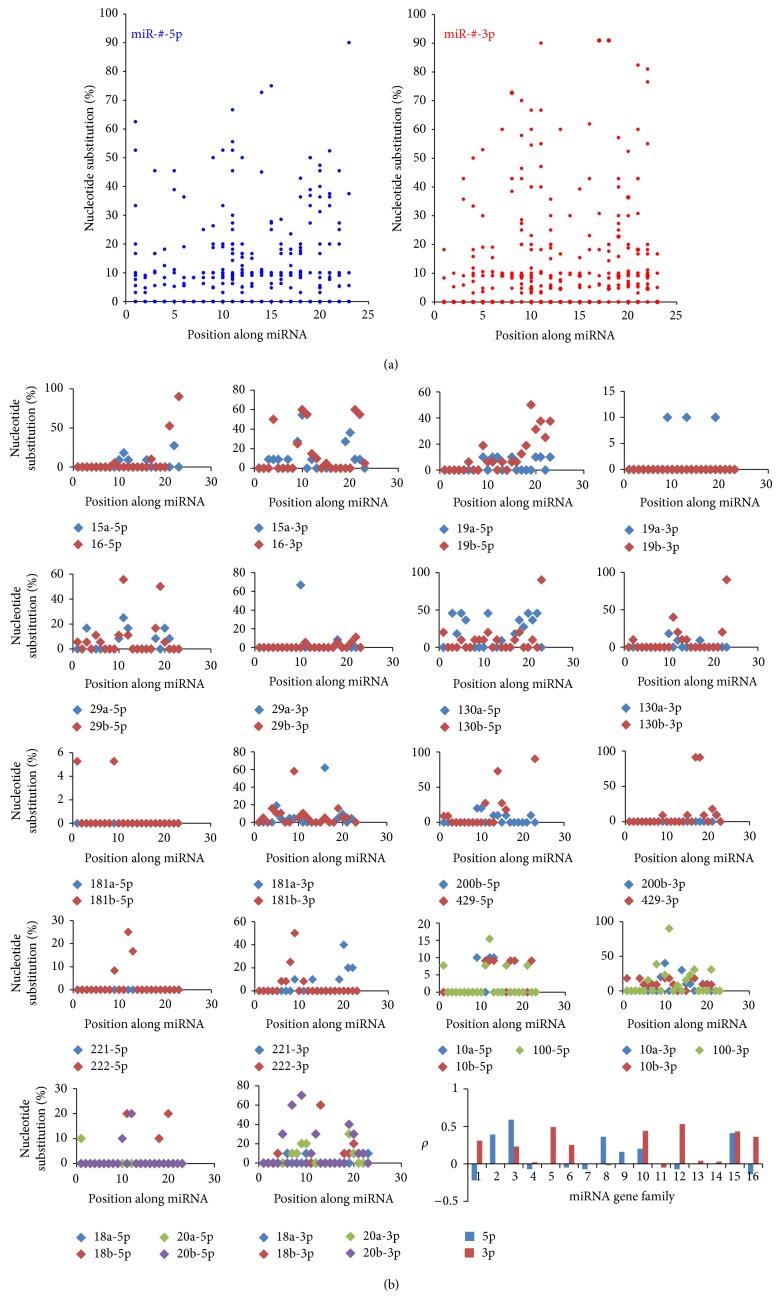
Scatter plots of miR-#-5p and miR-#-3p (a); homologous miRNAs and distribution of Spearman correlation coefficients (b). (a) Scatter plots of nucleotide substitution rates in the common miRNAs and their nondominant strands based on each position along miRNA (the conservation level was estimated based on nucleotide substitution rates along miRNA). (b) Scatter plots of nucleotide substitution between homologous miRNAs (miRNA gene family). miR-#-5p and miR-#-3p were compared and analyzed. The last figure indicates the distribution of Spearman correlation coefficient (*ρ*) across different miRNA gene families.

Although miRNAs are regarded as phylogenetically well-conserved small ncRNAs, different miRNAs, including homologous miRNAs, may show various evolutionary patterns (Figure S1) [15, 38]. Analysis of miR-#-5p and miR-#-3p revealed diverse variations in nucleotide composition (Figure S1). Compared to the dominant miRNAs, another strands showed higher levels of nucleotide diversity, haplotype diversity, and average number of nucleotide difference (Table 2). For example, let-7a-5p was highly conserved across the ten species, but let-7a-3p was associated with variation in the nucleotides. Generally, the dominant miRNAs were well-conserved, especially in the “seed sequences” (nucleotides 2–8), while nondominant miRNAs might display more variation in nucleotide composition (Figures S1C and S1D). Although both of them were reported as functional miRNAs existing at abundant levels in one or more species, 55.81% of miR-#-5p and miR-#-3p showed different levels of nucleotide divergence (Figure 2(a) and Table S3). The scatter plot analysis of the shared 43 miRNA genes revealed that both miR-#-5p and miR-#-3p were conserved (Figure 2(a)), with most sites showing minimal variation in nucleotide composition. Herein, 20 dominant miRNAs were identified as 5p-miRNA from 5p arm, and others (23 miRNAs) were identified as 3p-miRNA from 3p arm. We also analyzed the functional regions (seed sequences) of miRNAs, and only 4 pairs (9.30%) indicated difference (Table S3). The difference in average percentages from all the miR-#-5p and miR-#-3p was not significant (*Z* = −1.642, *P* > 0.05), and similar result could be detected based on the dominant miRNA (*Z* = −1.55, *P* > 0.05). Furthermore, although homologous miRNAs displayed close sequence, functional, and evolutionary relationships, no significant correlations were detected between most of homologous miRNAs (Figure 2(b) and Table S4).

**Figure 3:**
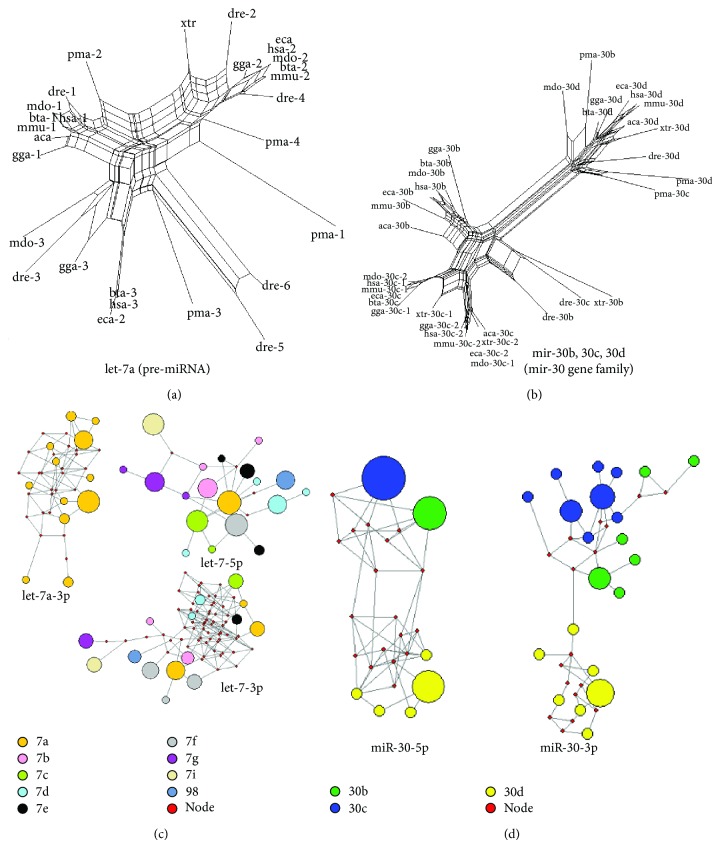
Examples of evolutionary patterns of different miRNAs. (a) Phylogenetic tree of let-7a. The tree is reconstructed using all the miRNA precursors, including multicopy pre-miRNAs. Multicopy pre-miRNAs are likely to be located in different clusters. (b) The phylogenetic tree of several members of mir-30 gene family (mir-30b, mir-30c, and mir-30d). The three members are split, and miR-30d shows a larger genetic distance with other members. (c) MJ networks of let-7a-3p, let-7-5p, and let-7-3p. Let-7a-3p is associated with nucleotide variation, although the other strand, let-7a-5p, is highly conserved. Similarly, let-7-5p from let-7 family also shows a simple network with several median vectors compared to let-7a-3p. Both let-7a-3p and let-7-3p show complex evolutionary networks with more median vectors. Members in let-7 gene family do not show clear module networks. The size of the circle shows the frequency of the miRNA haplotype (the specific miRNA sequence). (d) MJ networks of miR-30-5p and miR-30-3p from known miR-30b, miR-30c, and miR-30d sequences. Different miRNA members are likely to cluster together.

Phylogenetic trees and networks were reconstructed using pre-miRNAs and miRNAs from Figure S1, respectively (Figure 3). The phylogenetic tree of let-7a was split into three clusters, and each cluster contained pre-miRNAs from different animal species (Figure 3(a)). Compared to the tree of the single miRNA gene of let-7a, the phylogenetic tree of homologous mir-30b, mir-30c, and mir-30d could be split (Figure 3(b)). mir-30d showed larger genetic distance with mir-30b and mir-30c. The pma-mir-30b and pma-mir-30c were clustered with mir-30d, which indicates that these should be members of pma-mir-30d (Figure S1 and Figure 3). The evolutionary networks of miR-#-5p and miR-#-3p showed various patterns (Figures 3(c) and 3(d)). Different types of sequences (termed miRNA haplotypes) were classified with different frequencies. For example, let-7a-5p was highly conserved across the ten animal species, and only one specific sequence was identified. However, let-7a-3p was associated with high nucleotide variation and showed a complex evolutionary network (Figure S1A and Figure 3(c)). Compared to let-7, both evolutionary networks of miR-30-5p and miR-30-3p showed clear module networks based on miRNA members (Figure 3(d)).

### 3.3. Expression Analysis of miR-#-5p/miR-#-3p at the miRNA/isomiR Levels

**Figure 4:**
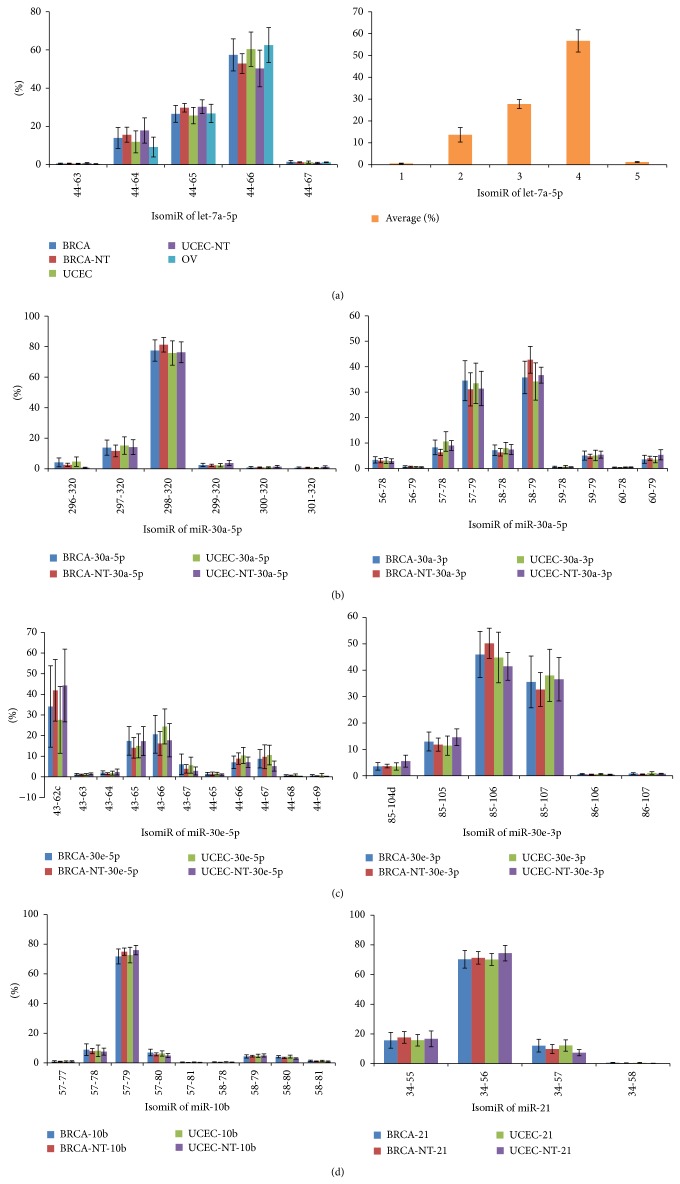
IsomiR expression patterns across different samples. IsomiR is presented here based on the location on chromosome (the detailed location distributions can be found in Tables S6, S7, and S8). The percentage shows the relative expression levels in the miRNA locus. The mean and standard deviation are presented in the figure. BRCA-NT or UCEC-NT shows normal samples that match tumor samples. (a) IsomiR expression patterns of let-7a-5p across the five kinds of samples. Similar distributions can be found across the different samples. The right bar chart indicates distribution of the mean percentage and standard deviation of the five kinds of samples. ((b)-(c)) IsomiR expression patterns of homologous miR-30a and miR-30e. Both of them can generate two kinds of abundant products (miR-#-5p and miR-#-3p). The two arms may show various isomiR expression patterns, but homologous miRNAs are likely to show similar expression patterns. (d) IsomiR expression patterns of miR-10b and miR-21.

**Figure 5:**
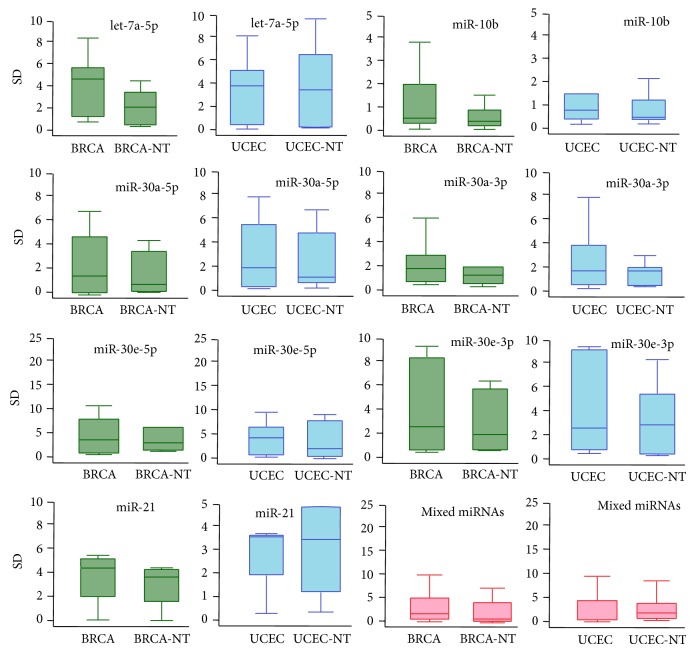
Box plots of miRNAs in BRCA and UCEC using standard deviation (SD). The box in green indicates the SD distribution of BRCA samples (BRCA and BRCA-NT), the box in blue indicates the SD distribution of UCEC samples (UCEC and UCEC-NT), and the box in pink indicates mixed miRNAs in different samples.

We analyzed available miRNA datasets of 2,144 patients or volunteers with women's diseases (BRCA, OV, or UCEC) and their relevant controls (Table S1). Following evolutionary analysis, several miRNAs were selected to perform expression analysis using these sequencing datasets. Generally, in the miRNA locus, only several isomiRs were dominantly expressed (Figure 4 and Tables S6, S7, and S8). Homologous miRNAs were likely to show similar isomiR expression pattern, such as miR-30a and miR-30e (Figure 4). Dominant miRNAs and their multiple isomiRs were present at abundant expression levels, while most of nondominant strands were not abundant. Abundantly expressed isomiRs were always near the most dominant isomiR sequence. Specifically, their 5′ or 3′ ends either were the same or differ at 1-2 nucleotides (Figure 4 and Tables S6, S7, and S8). The standard deviation (SD) of the average percentage of each isomiR showed diverse distributions (Figure 5 and Figures S2, S3, and S4). Different miRNAs showed different types of isomiRs with diverse expression distribution and SD (Figures 4 and 5 and Figures S2, S3, and S4). Abundantly expressed isomiRs were likely to be detected larger SD (Figure 4 and Figures S2 and S3), and similar SD distributions could be found between diseased and normal samples (Figure 5 and Figure S4). Generally, at the isomiR level, the average percentages of samples from disease patients would be involved in larger divergence than control samples, and similar results can be detected based on all miRNAs (Figure 5 and Figure S4).

### 3.4. Functional Analysis of miR-#-5p/miR-#-3p at the miRNA/isomiR Levels

Although miR-#-5p and miR-#-3p had different sequences and seed sequences, some common targets could be detected (Figure S5A). These miRNA pairs could bind different regions in UTR (untranslated regions) of target mRNAs, although the phenomenon was rare (larger amounts of specific targets could be detected). The common targets were more popular between the canonical miRNA sequences and their 5′ isomiRs, despite the fact that “seed shifting” could be detected between them (Figures S5B and S5C). There were about half of target mRNAs of 5′ isomiRs that were shared by the canonical miRNA sequences, although these 5′ isomiRs were involved in novel seed sequences via “seed shifting” events.

## 4. Discussion

### 4.1. Evolutionary Divergence between miRNAs from Different Arms

miRNAs have been widely regarded as a class of crucial negative regulatory molecules with important biological roles, especially for their roles in tumorigenesis. Based on the current annotated human miRNAs, similar numbers of 5p-miR and 3p-miR show well-conserved sequences across different species, although they are involved in inconsistent length distributions and nucleotide compositions, including multiple repetitive nucleotides (Figures 1(a)–1(c), Figure 2, and Table 1). This difference may be influenced by larger sample sizes. Simultaneously, mirtrons have been reported as alternative precursors for miRNA biogenesis in vertebrates [43], which may lead to the difference of nucleotide compositions because of nucleotide biases in mirtrons. There are 849 pairs that are identified as miR-#-5p and miR-#-3p, and significant difference in length distributions and nucleotide compositions is detected between the two arms (Figures 1(b)–1(d), Table 1, and Table S3). Evolutionary analysis shows that both dominant and nondominant miRNAs are conserved, although the nondominant miRNA is associated with more nucleotide variation across homologous miRNAs and different species [15]. Phylogenetic relationship shows that these multicopy pre-miRNAs are located in different clusters (Figure 3), which suggests the similar distributions of miRNA genes across different species. The well-conserved sequence contributes to stable miRNA-mRNA regulatory network, and simultaneously, the evolutionary process is also controlled by functional pressures. The two arms of pre-miRNA showed various evolutionary patterns via different levels of nucleotide substitutions and insertions/deletions (Figure S1, Figure 2, and Table S3), which may influence stem-loop structure of pre-miRNA (Table S5). However, both of the two arms are always well-conserved in the functional region, termed the “seed sequences” (Figure 3(a) and Table S3). These results suggest that both products from the two arms are regulatory molecules, although they always have various expression levels.

Homologous and clustered miRNAs are commonly found in miRNAs [44]. No significant relationships between these homologous miRNAs can be detected (Figure 2(b) and Table S4). These findings indicate relatively rapid evolutionary patterns between homologous miRNAs, especially between the less well-conserved nondominant strands (Figure 2(b)). Despite the possibility that these miRNAs have evolved from the common ancient miRNA gene, varied nucleotides in miRNAs, especially in the “seed sequences,” will generate novel miRNAs with novel candidate target mRNAs. Simultaneously, coevolution of miRNA and target mRNAs also contributes to the varied miRNAs across different species [45]. Taken together, homologous miRNAs may provide a method to generate novel miRNA genes via duplication events, and multicopy pre-miRNAs are probably transitional products. The driving force should be mainly derived from functional and evolutionary pressures, which largely contributes to the dynamic miRNAome, and enriches the potential relationships between different miRNAs.

### 4.2. Expression and Function between miRNAs from Different Arms

Similar to our previous studies [21, 46, 47], we found that only several isomiRs (always 1–3) are dominantly expressed, and others have lower expression rate (Figure 4 and Tables S6, S7, and S8). The interesting distributions are consistent in different individuals, including samples from patients with disease and healthy controls. The similar distributions suggest that isomiR expression patterns are always stable across different samples [21, 26]. The characteristics of these dominant isomiRs provide the possibility of imprecise cleavage of Drosha and Dicer through pre-miRNA processing and miRNA maturation processes. Indeed, due to the smaller size of miRNA sequence (~22 nt), degradation of hairpins may also be one factor that contributes to rare isomiRs [48]. Although the distribution of isomiR expression is similar across different samples, no significant correlations can be found between isomiR expression profiles of miR-#-5p and miR-#-3p (Figure 4). Simultaneously, various standard values of deviation can be found (Figure 5 and Figures S2, S3, and S4). Compared to control samples, samples from patients with disease may be involved in larger expression divergence across different samples (Figure 5). This suggests that a more flexible expression of isomiRs can be detected across different samples from patients with disease compared to control samples. Functional analysis showed that some common target mRNAs between miR-#-5p and miR-#-3p can be detected, although they have no different sequences and most target mRNAs are specific (Figure S5A). Simultaneously, more shared target mRNAs are obtained between the canonical miRNA and 5′ isomiRs despite being with “seed shifting” events (Figures S5B and S5C). The interesting results imply that multiple isomiRs may coordinately contribute to the specific biological processes by binding different regions in UTR. Moreover, 3′ addition events (isomiRs with additional nontemplate nucleotides in 3′ ends) are quite common in isomiRome, while no further analysis is performed in the present study based on the previous TCGA datasets. The phenomenon of 3′ additions may have versatile biological roles, including affecting target selection or miRNA stability [22, 24, 26, 49]. Collectively, analyzing multiple isomiRs and their expression patterns is the first step towards a systematic understanding of the miRNA world, including the genesis and regulatory roles of miRNAs.

miRNAs are likely to be members of miRNA gene families/clusters sharing high sequence similarity or close location distribution. These homologous/clustered miRNAs may have evolved from ancestor genes via part or tandem historic duplication events [15, 50–52]. Previous study reported that homologous miRNAs are likely to show similar isomiR expression patterns [47], and our results are consistent with this observation (Figure 4 and Table S7). The similarity in the expression patterns implies that the pre-miRNA processing and miRNA maturation processes should be derived from the ancestral gene, which may contribute to the potential interactions in the regulatory network [47]. Moreover, we found that deregulated miRNAs are likely to have different types of isomiRs (miR-30a, miR-30e, and miR-10b, Figure 4 and Tables S6, S7, and S8). These deregulated miRNAs have been reported in breast cancer [53, 54], and the moderate expression patterns can be detected. No enough evidence indicates that miRNA with moderate isomiR expression is likely to be abnormally expressed and contributes to abnormal biological roles. More studies, especially for experimental validation, are needed to further study the small noncoding RNAs at the isomiR level.

### 4.3. Selection of 5p and 3p or Switching between the Two Arms in miRNAome/isomiRome

The phenomenon of arm selection shows that miRNAs may be derived from different arms, and the arm switching phenomenon suggests that the two arms may also show dynamic expression patterns. miRNAs from the two arms (they can form miRNA:miRNA duplex) always show different evolutionary patterns and also have various expression levels and isomiR expression patterns. Most of pre-miRNAs only produce one dominant and one rare miRNAs in specific samples, although the expression rate of the two miRNAs may be changed in other samples (arm switching phenomenon). Indeed, the two arms of many pre-miRNAs are conserved (especially in “seed sequences”), providing the possibility to be regulatory molecules, and the arm switching phenomenon further enriches the dynamic miRNAome by controlling miRNA expression profiles to adapt to functional and/or evolutionary needs. Expression and evolution patterns in miR-#-5p and miR-#-3p are relatively independent, and they are prone to regulate different targets. Based on the phenomena of arm selection or arm switching, the dynamic miRNAome also represents the multiple and dynamic isomiRome at the isomiR level. These isomiRs provide more information towards further understanding of miRNAs, in that isomiR expression patterns may indicate the characteristics of pre-miRNA processing and miRNA maturation processes. Thus it is worth exploring the biological roles of miRNAs at the isomiR level and the origin of miRNAs (5p or 3p) and related miRNAs based on miRNA gene family/cluster. Taken together, the arm selection and/or arm switching may be an important method to regulate miRNAome and isomiRome, and the dynamic miRNA and isomiR expression profiles will adapt to functional and/or evolutionary pressures.

## Supplementary Material

Figure S1: showed that examples of nucleotide divergence between different miRNAs, including 5p-miRNA and 3p-miRNA, and miR-#-5p and miR-#-3p.
Figure S2-S4: showed box plots of miRNAs between different samples using standard deviation (SD), and Figure S5 presented examples of functional analysis.
Table S1: listed selected small RNA sequencing datasets from the TCGA database.
Table S2: showed the common miRNAs in the ten animal species.
Table S3: presented Wilcoxon signed-rank test of miR-#-5p and miR-#-3p.
Table S4: showed spearman correlation coefficient between homologous miRNAs.
Table S5: presented the free energies of some pre-miRNAs.
Table S6-S8: showed isomiR expression distributions of let-7a-5p, homologous miR-30a and miR-30e, miR-10b and miR-21 across all samples.

## Figures and Tables

**Table 1:** Frequency of nucleotide compositions between all human miRNAs from different arms.

Nucleotides	5p-miRNA (%)	3p-miRNA (%)	miR-#-5p (%)	miR-#-3p (%)	*χ* ^2^, *P*
AA	18.38	18.13	17.98^c^	17.09	
UU	22.21	24.39	22.59	25.68	
CC	17.39	30.64	17.80	36.06	
GG	42.02	26.84	41.63	21.17	
Total	**100**	**100**	**100**	**100**	21.31, 0.01
AAA	16.26^a^	17.77	14.39^d^	16.21	
UUU	20.83	23.41	21.36	24.98	
CCC	15.38	33.80	15.40	40.90	
GGG	47.53	25.02	48.85	17.91	
Total	**100**	**100**	**100**	**100**	42.74, 0.00
AAAA	17.72^b^	19.87	14.97^e^	17.95	
UUUU	21.44	22.03	20.38	22.76	
CCCC	8.97	34.99	8.60	45.83	
GGGG	51.86	23.11	56.05	13.46	
Total	**100**	**100**	**100**	**100**	81.10, 0.00

The percentage is estimated based on frequency in all the 5p- or 3p-miRNAs, all the miR-#-5p or miR-#-3p. ^a^A significant difference in the triple repetitive nucleotides can be detected between 5p-miRNA and 3p-miRNA (*χ* ^2^ = 14.82, *P* < 0.01), ^b^a significant difference in the four repetitive nucleotides can be detected between 5p-miRNA and 3p-miRNA (*χ* ^2^ = 26.71, *P* < 0.01), ^c^a significant difference in the double repetitive nucleotides can be detected between miR-#-5p and miR-#-3p (*χ* ^2^ = 13.21, *P* < 0.01), ^d^a significant difference in the triple repetitive nucleotides can be detected between miR-#-5p and miR-#-3p (*χ* ^2^ = 26.89, *P* < 0.01), and ^e^a significant difference in the four repetitive nucleotides can be detected between miR-#-5p and miR-#-3p (*χ* ^2^ = 52.17, *P* < 0.01).

**Table 2:** Nucleotide diversity (*π*), haplotype diversity (Hd), and average number of nucleotide differences (*k*) of different miRNA populations.

miRNA	miRNA-#-5p			miR-#-3p		
	*π*	Hd	*k*	*π*	Hd	*k*
let-7a	0.00	0.00	—	0.18 ± 0.01	0.86 ± 0.05	3.69
Total (let-7 family, 76, 45)	0.12 ± 0.01	0.92 ± 0.01	2.60	0.25 ± 0.01	0.93 ± 0.01	4.90
miR-30b	0.00	0.00	—	0.14 ± 0.04	0.83 ± 0.13	2.86
miR-30c	0.00	0.00	—	0.11 ± 0.03	0.80 ± 0.08	2.09
miR-30d	0.04 ± 0.01	0.58 ± 0.16	0.91	0.12 ± 0.03	0.77 ± 0.13	2.35
Total (part mir-30 family)	0.08 ± 0.01	0.52 ± 0.08	1.83	0.28 ± 0.02	0.93 ± 0.02	5.26

These parameters are estimated according to Figure S1.

## Abbreviations

miRNA: MicroRNA
ncRNA: Noncoding RNA
pri-miRNA: Primary miRNA
pre-miRNA: Precursor miRNA
miRNA^∗^: miRNA star
SNP: Single nucleotide polymorphism
MJ: Median-joining
TCGA: The Cancer Genome Atlas
BRCA: Breast cancer
OV: Ovarian serous cystadenocarcinoma
UCEC: Uterine corpus endometrial carcinoma
SD: Standard deviation
pma: *Petromyzon marinus*
dre: *Danio rerio*
xtr: *Xenopus tropicalis*
aca: *Anolis carolinensis*
gga: *Gallus gallus*
*eca*: *Equus caballus*
bta: *Bos taurus*
*mdo*: *Monodelphis domestica*
*mmu*: *Mus musculus*
hsa: *Homo sapiens*.
