# Supplementary material for: Evolutionary and Expression Analysis of miR-#-5p and miR-#-3p at the miRNAs/isomiRs Levels
Source: Biomed Res Int. 2015 May 5;2015:168358. doi: 10.1155/2015/168358 (PMC4436453; doi:10.1155/2015/168358)
Supplement: Supplementary file 1 — Figure S1: showed that examples of nucleotide divergence between different miRNAs, including 5p-miRNA and 3p-miRNA, and miR-#-5p and miR-#-3p. Figure S2-S4: showed box plots of miRNAs between different samples using standard deviation (SD), and Figure S5 presented examples of functional analysis. Table S1: listed selected small RNA sequencing datasets from the TCGA database. Table S2: showed the common miRNAs in the ten animal species. Table S3: presented Wilcoxon signed-rank test of miR-#-5p and miR-#-3p. Table S4: showed spearman correlation coefficient between homologous miRNAs. Table S5: presented the free energies of some pre-miRNAs. Table S6-S8: showed isomiR expression distributions of let-7a-5p, homologous miR-30a and miR-30e, miR-10b and miR-21 across all samples. [file 168358.f1.doc]

**Evolutionary and expression analysis of miR-#-5p and miR-#-3p at the miRNAs/isomiRs levels**

Li Guo^1^*, Jiafeng Yu^2^, Hao Yu^1^, Yang Zhao^1^, Shujie Chen^1^, Changqing Xu^1^, Feng Chen^1^

^1^ Department of Epidemiology and Biostatistics, School of Public Health, Nanjing Medical University, Nanjing, 211166, China

^2^ Shandong Provincial Key Laboratory of Functional Macromolecular Biophysics, Institute of Biophysics, Dezhou University, Dezhou, 253023,China

* To whom correspondence should be addressed.

E-mail addresses:

LG: [gl8008@163.com](mailto:gl8008@163.com), [lguo@njmu.edu.cn](mailto:lguo@njmu.edu.cn)

**Supplemental materials**

**
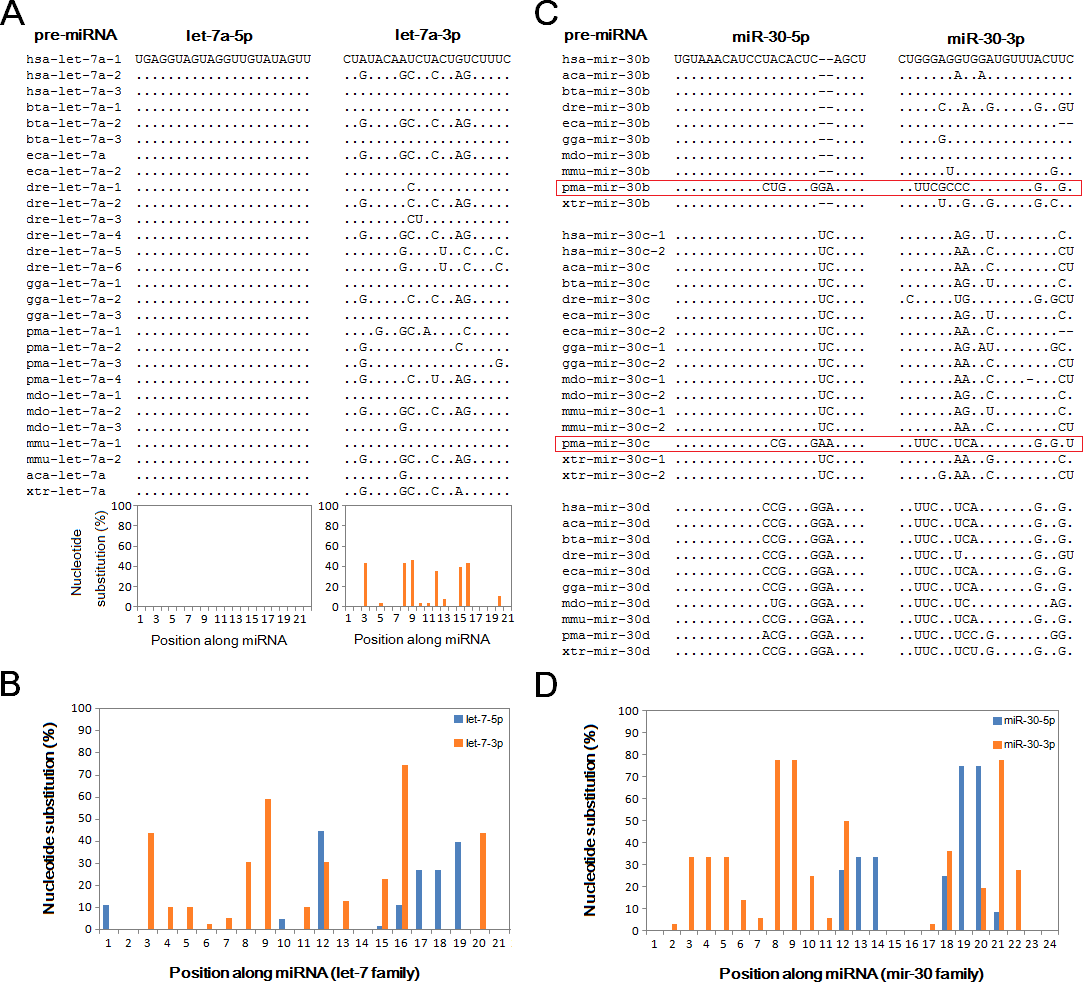
**

**Figure S1.** Examples of nucleotide divergence between different miRNAs, including 5p-miRNA and 3p-miRNA, and miR-#-5p and miR-#-3p.

(A) Sequence diversities of let-7a-5p and let-7a-3p. All the pre-miRNAs of let-7a, including multicopy pre-miRNAs, were analyzed. (B) Nucleotide substitution rate in each position based on all known let-7-5p and let-7-3p in the let-7 gene family. Compared to the well-conserved let-7-5p, let-7-3p is more likely to be associated with nucleotide variation across different let-7 members and animal species. (C) Sequence diversities of miR-30b, miR-30c and miR-30d. According to the sequence alignment results, pma-miR-30b and pma-miR-30c should be characterized as members of miR-30d. The phylogenetic network analysis also supports the result, and the two miRNAs are clustered together with miR-30d, Fig. 3B and 3D. (D) Nucleotide substitution rate in each position based on (C). miR-30-5p sequences are well-conserved across different homologous miRNAs and animal species, while miR-30-3p sequences are more involved with nucleotide variation.

**Figure S2.** Box plots of miRNAs between different samples using standard deviation (SD).

Box plots were estimated based on isomiRs (locations) of different miRNAs. The box in green indicates the SD distribution of samples from patients with disease (BRCA and UCEC), and the box in yellow indicates the SD distribution of normal samples (BRCA-NT and UCEC-NT), and the box in pink indicates mixed miRNAs based on the most and the secondary dominant isomiR.

**Figure S3.** Box plots of miRNAs using standard deviation (SD).

Box plots were estimated based on isomiRs (locations) of different miRNAs without considering different samples (green box), and box plot was estimated based on each miRNA (red box).

**Figure S4.** Box plots of miRNAs using standard deviation (SD).

Box plots are estimated based on different samples (green box) without considering detailed isomiRs, and box plot is estimated based on different samples using mixed miRNAs (yellow box). The BRCA, UCEC and their control samples are presented together based on the consistent SD scales.

**Figure S5.** Examples of functional analysis.

(A) Functional analysis between miR-#-5p and miR-#-3p; (B-C) indicates the potential functional relationships between the canonical miRNA sequences and their isomiRs with the novel 5’ ends and seed sequences.

**Table S1.** Selected small RNA sequencing datasets from the TCGA database.

| **Women Disease** | **TN (tumor, matched normal)** | **NT (normal, matched tumor)** | **Total** |
| --- | --- | --- | --- |
| BRCA | 683 | 87 | 770 |
| OV | 970 | － | 970 |
| UCEC | 383 | 21 | 404 |
| Total | 2,036 | 108 | 2,144 |

“－” indicates that the small RNA sequencing datasets of OV-NT are not available in the TCGA database.

**Table S2.** The common miRNAs in the ten animal species.

| **Gene family** | **miRNA members** | **Gene family** | **miRNA members** |
| --- | --- | --- | --- |
| let-7 | *let-7a*, let-7b, let-7c, let-7d, let-7e, let-7f, let-7g, let-7i, miR-98 | mir-126 | *miR-126* |
| mir-8 | miR-141, miR-200a, *miR-200b*, miR-200c, *miR-429* | mir-128 | *miR-128* |
| mir-9 | *miR-9* | mir-130 | *miR-130a*, *miR-130b*, miR-301a, miR-301b |
| mir-10^①^ | *miR-10a*, *miR-10b*, miR-99a, miR-99b, *miR-100*, miR-125a, miR-125b | mir-133 | *miR-133a*, miR-133b |
| mir-15 | *miR-15a*, miR-15b, *miR-16*, miR-195 | mir-137 | *miR-137* |
| mir-17 | miR-17, *miR-18a*, *miR-18b*, *miR-20a*, *miR-20b*, miR-93, miR-106a, miR-106b | mir-138 | *miR-138* |
| mir-19 | *miR-19a*, *miR-19b* | mir-140 | *miR-140* |
| mir-22 | *miR-22* | mir-144 | *miR-144* |
| mir-23 | miR-23a, *miR-23b* | mir-153 | *miR-153* |
| mir-24 | *miR-24*, miR-3074 | mir-181 | *miR-181a*, *miR-181b*, miR-181c, miR-181d |
| mir-25 | miR-25, *miR-92a*, miR-92b | mir-184 | *miR-184* |
| mir-26 | *miR-26a*, miR-26b | mir-204 | *miR-204*, miR-211 |
| mir-27 | miR-27a, *miR-27b* | mir-217 | *miR-217* |
| mir-29 | *miR-29a*, *miR-29b*, miR-29c | mir-218 | *miR-218* |
| mir-30 | miR-30a, *miR-30b*^②^, *miR-30c*^②^, *miR-30d*, miR-30e | mir-221 | *miR-221*, *miR-222* |
| mir-103 | miR-103a, miR-103b, miR-107 (*miR-103*)^③^ | mir-99^①^ | miR-99a, miR-99b, *miR-100* |

miRNA members are presented here according to human miRNAs. The italic and underlined miRNAs are shared by the 10 animal species (43 miRNAs from 31 miRNA gene families). ^①^ indicates that mir-99a, mir-99b and mir-100 are also termed mir-99 gene family. ^②^ indicates that the two miRNAs are identified as common miRNAs according to annotated known miRNAs, but pma-miR-30b and pma-miR-30c should be characterized as pma-miR-30d (Figure 2 and Figure 3).  ^③^indicates that the miRNA is shared by the ten test animal species, although multiple homologous members are identified in other specific species.

**Table S3.**Wilcoxon signed-rank test of miR-#-5p and miR-#-3p.

| **miRNA**  **(5p & 3p)** | **The miRNA sequence** | | **Seed sequences (nucleotides 2-8)** | |
| --- | --- | --- | --- | --- |
|  | ***Z*** | ***P*** | ***Z*** | ***P*** |
| let-7a | -3.27 | 0.00 | -1.72 | 0.09 |
| miR-200b | 2.23 | 0.03 | － | － |
| miR-429 | 1.98 | 0.047 | 1.00 | 0.32 |
| miR-9 | -0.51 | 0.61 | 0.00 | 1.00 |
| miR-15a | -1.74 | 0.08 | -2.00 | 0.045 |
| miR-16 | -2.53 | 0.01 | -1.00 | 0.32 |
| miR-10a | -1.73 | 0.08 | － | － |
| miR-10b | -2.63 | 0.01 | -2.19 | 0.03 |
| miR-100 | -2.29 | 0.02 | -1.41 | 0.16 |
| miR-18a | -2.45 | 0.01 | -1.00 | 0.32 |
| miR-18b | -1.62 | 0.11 | -1.73 | 0.08 |
| miR-20a | -2.17 | 0.03 | -1.41 | 0.16 |
| miR-20b | -2.59 | 0.01 | -1.41 | 0.16 |
| miR-19a | 1.41 | 0.16 | － | － |
| miR-19b | 3.64 | 0.00 | 1.00 | 0.32 |
| miR-22 | 1.62 | 0.11 | 1.08 | 0.28 |
| miR-23b | 2.00 | 0.045 | 1.00 | 0.32 |
| miR-24 | 3.27 | 0.00 | 1.41 | 0.16 |
| miR-92a | 2.53 | 0.01 | 1.41 | 0.16 |
| miR-26a | -3.64 | 0.00 | -1.96 | 0.05 |
| miR-27b | 2.82 | 0.00 | 1.00 | 0.32 |
| miR-29a | 1.53 | 0.13 | 1.00 | 0.32 |
| miR-29b | -2.34 | 0.02 | 1.72 | 0.09 |
| miR-30d | -1.69 | 0.09 | － | － |
| miR-103 | 0.87 | 0.39 | 1.41 | 0.16 |
| miR-126 | -0.17 | 0.87 | 1.00 | 0.32 |
| miR-128 | 3.40 | 0.00 | 1.41 | 0.16 |
| miR-130a | 2.87 | 0.00 | 1.96 | 0.049 |
| miR-130b | 0.59 | 0.55 | 0.58 | 0.56 |
| miR-133a | 0.58 | 0.56 | － | － |
| miR-137 | 0.58 | 0.56 | 1.00 | 0.32 |
| miR-138 | -3.27 | 0.00 | -1.72 | 0.09 |
| miR-140 | -1.41 | 0.16 | -1.00 | 0.32 |
| miR-144 | 0.60 | 0.55 | － | － |
| miR-153 | 0.92 | 0.36 | － | － |
| miR-181a | -2.98 | 0.00 | -1.72 | 0.08 |
| miR-181b | -3.09 | 0.00 | -1.96 | 0.049 |
| miR-184 | 0.96 | 0.34 | － | － |
| miR-204 | -2.97 | 0.00 | -1.72 | 0.09 |
| miR-217 | -1.92 | 0.05 | － | － |
| miR-218 | -2.49 | 0.01 | -1.41 | 0.16 |
| miR-221 | 2.44 | 0.01 | － | － |
| miR-222 | 1.07 | 0.28 | 1.72 | 0.09 |

**Table S4.** Spearman correlation coefficient between homologous miRNAs.

| **miRNA gene family** | **miRNA members** | | | | **ρ_s_ (5p,3p)** | ***P* (5p,3p)** |
| --- | --- | --- | --- | --- | --- | --- |
| mir-15 | miR-15a, miR-16 |  |  |  | -0.22,0.31 | 0.33,0.15 |
| mir19 | miR-19a, miR-19b |  |  |  | 0.39,－ | 0.06,－ |
| mir-29 | miR-29a, miR-29b |  |  |  | 0.59,0.23 | 0.00,0.29 |
| mir-130 | miR-130a, miR-130b |  |  |  | -0.07,0.02 | 0.74,0.92 |
| mir-181 | miR-181a, miR-181b |  |  |  | －,0.49 | －,0.02 |
| mir-8 | miR-200b, miR-429 |  |  |  | -0.05,0.25 | 0.86,0.24 |
| mir-221 | miR-221, miR-222 |  |  |  | -0.07,－ | 0.76,－ |
| mir-10 | miR-10a, miR-10b |  |  |  | 0.36,-0.02 | 0.09,0.94 |
| mir-10 | miR-10a, miR-100 |  |  |  | 0.16,-0.00 | 0.46,0.99 |
| mir-10 | miR-10b, miR-100 |  |  |  | 0.20,0.44 | 0.37,0.04 |
| mir-17 | miR-18a, miR-18b |  |  |  | －,-0.05 | －,0.82 |
| mir-17 | miR-20a, miR-20b |  |  |  | -0.07,0.53 | 0.77,0.01 |
| mir-17 | miR-18a, miR-20a |  |  |  | －,0.04 | －,0.87 |
| mir-17 | miR-18a, miR-20b |  |  |  | －,0.03 | －,0.91 |
| mir-17 | miR-18b, miR-20a |  |  |  | 0.41,0.43 | 0.05,0.04 |
| mir-17 | miR-18b, miR-20b |  |  |  | -0.14,0.36 | 0.52,0.09 |

“－” indicates that one member is not involved in varied nucleotides across the ten animal spices.

**Table S5.** The free energies of some pre-miRNAs.

| **miRNA** | **Minimum free energy (kcal/mol)** | **Free energy (kcal/mol)** | | | | **Frequency of the MFE structure (%)** | **Ensemble diversity** |
| --- | --- | --- | --- | --- | --- | --- | --- |
| hsa-let-7a-1 | -33.20 |  | -34.22 |  |  | 19.13 | 5.95 |
| hsa-let-7a-2 | -25.20 |  | -26.36 |  |  | 15.17 | 4.45 |
| hsa-let-7a-3 | -34.10 |  | -34.96 |  |  | 24.64 | 3.01 |
| hsa-mir-30c-1 | -34.70 |  | -35.71 |  |  | 19.48 | 6.87 |
| hsa-mir-30c-2 | -25.40 |  | -25.90 |  |  | 44.16 | 1.32 |
| xtr-mir-30c-1 | -39.20 |  | -40.11 |  |  | 22.68 | 2.89 |
| xtr-mir-30c-2 | -31.80 |  | -32.07 |  |  | 64.40 | 3.07 |
| gga-mir-30c-1 | -37.40 |  | -38.64 |  |  | 13.26 | 3.94 |
| gga-mir-30c-2 | -26.50 |  | -27.24 |  |  | 29.99 | 3.16 |

**Table S6.** IsomiR expression distributions of let-7a-5p across all samples.

| **Let-7a-5p** | **BRCA** | **UCEC** | **OV** | **BRCA-NT** | **UCEC-NT** |
| --- | --- | --- | --- | --- | --- |
| 8244-8263* | 0.52±0.21 | 0.48±0.22 | 0.29±0.17 | 0.57±0.14 | 0.74±0.31 |
| 8244-8264 | 13.93±5.49 | 11.87±5.74 | 9.21±5.18 | 15.59±3.94 | 17.83±6.57 |
| 8244-8265 | 26.54±4.41 | 25.63±4.30 | 26.75±4.74 | 29.73±2.26 | 30.26±3.60 |
| 8244-8266 | 57.35±8.38 | 60.36±8.98 | 62.47±9.15 | 52.85±5.18 | 50.26±9.61 |
| 8244-8267 | 1.47±0.68 | 1.26±0.57 | 1.16±0.19 | 1.20±0.26 | 0.84±0.36 |
| Total (mean) | 99.81 | 99.60 | 99.88 | 99.94 | 99.93 |

The isomiR is presented here using mean±sd (standard deviation). The location of isomiRs of let-7a is presented using the location distribution on human chromosome. * indicates that the location should be 96,938,244-96,938,263 on chromosome 9(+). Only those isomiRs with higher expression rate (near 0.50%) are presented here.

**Table S7.** IsomiR expression distributions of homologous miR-30a and miR-30e.

| **miR-30a-5p** | **BRCA-30a-5p** | **BRCA-NT-30a-5p** | **UCEC-30a-5p** | **UCEC-NT-30a-5p** |
| --- | --- | --- | --- | --- |
| 3296-3320^a^ | 4.21±2.89 | 2.57±1.10 | 4.66±3.05 | 0.68±0.34 |
| 3297-3320 | 13.81±4.98 | 11.67±3.82 | 15.20±5.68 | 14.14±4.88 |
| 3298-3320 | 77.44±6.96 | 81.25±4.78 | 75.79±7.97 | 76.33±6.81 |
| 3299-3320 | 2.49±1.07 | 2.29±0.74 | 2.33±1.08 | 3.77±1.72 |
| 3300-3320 | 0.89±0.61 | 0.92±0.28 | 0.90±0.41 | 1.45±0.77 |
| 3301-3320 | 0.67±0.49 | 0.77±0.26 | 0.64±0.33 | 1.26±0.72 |
| Total (mean) | 99.51 | 99.47 | 99.52 | 97.63 |
| **miR-30a-3p** | **BRCA-30a-3p** | **BRCA-NT-30a-3p** | **UCEC-30a-3p** | **UCEC-NT-30a-3p** |
| 3256-3278^b^ | 3.40±1.26 | 3.11±0.86 | 3.13±1.18 | 2.92±0.93 |
| 3256-3279 | 0.70±0.37 | 0.68±0.22 | 0.49±0.26 | 0.47±0.21 |
| 3257-3278 | 8.31±2.89 | 6.31±1.21 | 10.61±3.84 | 9.04±1.93 |
| 3257-3279 | 34.51±7.87 | 31.09±6.53 | 33.48±7.94 | 31.42±6.76 |
| 3258-3278 | 7.19±2.08 | 6.35±1.48 | 8.02±2.25 | 7.45±1.97 |
| 3258-3279 | 35.78±6.40 | 42.73±5.25 | 34.18±7.30 | 36.69±3.15 |
| 3259-3278 | 0.60±0.30 | 0.34±0.10 | 0.73±0.44 | 0.58±0.14 |
| 3259-3279 | 5.06±1.78 | 4.77±0.86 | 5.14±2.08 | 5.43±1.37 |
| 3260-3278 | 0.38±0.16 | 0.28±0.07 | 0.44±0.18 | 0.51±0.14 |
| 3260-3279 | 3.61±1.58 | 4.03±0.83 | 3.49±1.27 | 5.39±2.00 |
| Total (mean) | 99.54 | 99.69 | 99.71 | 99.90 |
| **miR-30e-5p** | **BRCA-30e-5p** | **BRCA-NT-30e-5p** | **UCEC-30e-5p** | **UCEC-NT-30e-5p** |
| 43-62^c^ | 34.11±19.74 | 41.92±14.92 | 27.59±16.21 | 44.28±17.65 |
| 43-63 | 1.18±0.45 | 1.04±0.27 | 1.15±0.41 | 1.47±0.49 |
| 43-64 | 2.05±0.82 | 1.51±0.50 | 1.81±0.96 | 2.37±1.42 |
| 43-65 | 17.42±6.91 | 14.08±4.97 | 15.01±5.81 | 17.31±7.05 |
| 43-66 | 20.64±9.17 | 16.20±5.74 | 24.42±8.49 | 17.75±8.10 |
| 43-67 | 6.11±4.94 | 3.87±2.07 | 5.65±3.89 | 2.87±1.95 |
| 44-65 | 1.48±0.70 | 1.50±0.83 | 1.59±0.64 | 1.12±0.34 |
| 44-66 | 7.06±3.06 | 8.86±2.76 | 10.38±3.90 | 7.13±2.49 |
| 44-67 | 8.74±4.44 | 9.75±5.78 | 10.55±4.76 | 5.20±2.43 |
| 44-68 | 0.65±0.38 | 0.56±0.30 | 0.84±0.58 | 0.23±0.11 |
| 44-69 | 0.62±0.50 | 0.37±0.12 | 0.76±0.87 | 0.25±0.05 |
| Total (mean) | 100.06@ | 99.66 | 99.75 | 99.98 |
| **miR-30e-3p** | **BRCA-30e-3p** | **BRCA-NT-30e-3p** | **UCEC-30e-3p** | **UCEC-NT-30e-3p** |
| 85-104^d^ | 3.56±1.43 | 3.70±0.68 | 3.53±1.39 | 5.54±2.27 |
| 85-105 | 12.97±3.58 | 11.79±2.50 | 11.43±3.67 | 14.61±3.18 |
| 85-106 | 45.98±8.74 | 50.20±5.75 | 44.83±9.60 | 41.50±5.31 |
| 85-107 | 35.57±9.83 | 32.70±6.45 | 38.05±9.90 | 36.60±8.23 |
| 86-106 | 0.64±0.21 | 0.51±0.14 | 0.66±0.23 | 0.47±0.13 |
| 86-107 | 0.83±0.33 | 0.56±0.13 | 1.06±0.49 | 0.75±0.17 |
| Total (mean) | 99.55 | 99.46 | 99.56 | 99.47 |

^a^ indicates that the location should be 72,113,296-72,113,320 on chromosome 6(-), ^b^ indicates that the location should be 72,113,256-72,113,278 on chromosome 6(-), ^c^ indicates that the location should be 41,220,043-41,220,062 on chromosome 1(+), and ^d^ indicates that the location should be 41,220,085-41,220,104 on chromosome 1(+). @ indicates that the total mean is larger than 100, which is mainly derived from expression divergence between different samples.

**Table S8.** IsomiR expression distributions of miR-10b and miR-21.

| **miR-10b** | **BRCA-10b** | **BRCA-NT-10b** | **UCEC-10b** | **UCEC-NT-10b** |
| --- | --- | --- | --- | --- |
| 57-77 | 0.95±0.52 | 0.93±0.24 | 0.94±0.48 | 1.03±0.44 |
| 57-78 | 8.92±3.94 | 7.98±1.74 | 8.20±3.88 | 7.63±2.38 |
| 57-79 | 71.73±5.05 | 74.97±2.46 | 72.62±5.25 | 76.02±3.15 |
| 57-80 | 7.05±2.17 | 5.77±1.01 | 6.37±1.76 | 4.99±1.33 |
| 57-81 | 0.39±0.30 | 0.29±0.09 | 0.39±0.26 | 0.28±0.14 |
| 58-78 | 0.48±0.29 | 0.41±0.10 | 0.47±0.34 | 0.44±0.19 |
| 58-79 | 4.41±1.05 | 4.58±0.61 | 4.81±1.07 | 5.03±1.07 |
| 58-80 | 4.16±0.74 | 3.39±0.50 | 4.31±0.96 | 2.98±0.47 |
| 58-81 | 1.38±0.50 | 1.11±0.23 | 1.31±0.46 | 0.94±0.34 |
| Total (mean) | 99.47 | 99.43 | 99.42 | 99.34 |
| **miR-21** | **BRCA-21** | **BRCA-NT-21** | **UCEC-21** | **UCEC-NT-21** |
| 34-55 | 15.69±5.32 | 17.60±3.96 | 15.74±3.88 | 16.68±5.31 |
| 34-56 | 70.30±5.91 | 71.27±4.24 | 70.15±4.01 | 74.44±5.27 |
| 34-57 | 12.09±4.29 | 9.83±3.06 | 12.20±3.83 | 7.34±2.07 |
| 34-58 | 0.56±0.31 | 0.35±0.15 | 0.53±0.31 | 0.20±0.10 |
| Total (mean) | 98.64 | 99.05 | 98.62 | 98.66 |
